# Strip-Mode Microwave Staring Correlated Imaging with Self-Calibration of Gain–Phase Errors

**DOI:** 10.3390/s19051079

**Published:** 2019-03-03

**Authors:** Rui Xia, Yuanyue Guo, Weidong Chen, Dongjin Wang

**Affiliations:** Key Laboratory of Electromagnetic Space Information, Chinese Academy of Sciences, University of Science and Technology of China, Hefei 230026, China; xrke928@mail.ustc.edu.cn (R.X.); wdchen@ustc.edu.cn (W.C.); wangdj@ustc.edu.cn (D.W.)

**Keywords:** microwave staring correlated imaging (MSCI), gain–phase errors, strip, self-calibration

## Abstract

Microwave staring correlated imaging (MSCI) can realize super resolution imaging without the limit of relative motion with the target. However, gain–phase errors generally exist in the multi-transmitter array, which results in imaging model mismatch and degrades the imaging performance considerably. In order to solve the problem of MSCI with gain–phase error in a large scene, a method of MSCI with strip-mode self-calibration of gain–phase errors is proposed. The method divides the whole imaging scene into multiple imaging strips, then the strip target scattering coefficient and the gain–phase errors are combined into a multi-parameter optimization problem that can be solved by alternate iteration, and the error estimation results of the previous strip can be carried into the next strip as the initial value. All strips are processed in multiple rounds, and the gain–phase error estimation results of the last strip can be taken as the initial value and substituted into the first strip for the correlated processing of the next round. Finally, the whole imaging in a large scene can be achieved by multi-strip image splicing. Numerical simulations validate its potential advantages to shorten the imaging time dramatically and improve the imaging and gain–phase error estimation performance.

## 1. Introduction

Radar imaging technology [1,2] has enabled radars to have the ability to obtain a panoramic image of an observation scene, which has been widely used in military warning, disaster detection and other fields. In these application scenarios, long-term continuous monitoring of large areas is an important application requirement.

Synthetic aperture radar (SAR) has high azimuth resolution imaging ability by forming large virtual synthetic aperture through relative motion between the target and radar, but its long revisiting period means that it cannot be applied to the staring imaging [3,4].

Traditional real aperture microwave staring imaging has the characteristics of high real-time, but limited by the actual aperture of the antenna; its azimuth resolution is low, so it is difficult to achieve high resolution imaging. As a new staring imaging method, microwave staring correlated imaging (MSCI) [5,6,7] can realize super resolution imaging without the limit of the target relative motion. The essence of MSCI is to construct a temporal–spatial stochastic radiation field in the imaging region, which is typically realized by a multi-transmitter array transmitting independent stochastic waveforms [5,6] such as the signals with random amplitude and frequency between different pulses. The radiation field interacts with the target so that the target scattering points at different locations scatter the independent time-varying echoes. Finally, the target information can be obtained by the correlated imaging process between the echoes and the preset radiation field. In [7], two point targets in a small scene are imaged by outfield experiments based on MSCI. Accurate imaging is based on the premise of accurate preset radiation field. However, gain–phase errors generally exist in the multi-transmitter array, so there is a deviation between the actual radiation field and the preset radiation field that is calculated based on transmitted waveform, which results in the imaging model mismatch and degrades the imaging performance considerably. In [8,9], the methods are propose for model mismatch in radar coincidence imaging (RCI), but the gain–phase error model was not analysed.

The studies on calibration of gain–phase error mainly focus on a radar system with multiple transmissions or multiple receptions, including the field of angle estimation of array signals [10,11] and radar imaging [12,13,14,15,16,17]. In [10], a method based on eigenstructure is proposed for simultaneously estimating the direction of arrival(DOA) and the unknown (or imprecisely known) gain and phase parameters, which applies to arrays with arbitrary sensor geometries. The method is based on the eigendecomposition of the sample covariance matrix of the vector of received signals. In [11], an estimation of signal parameters via a rotational invariance techniques (ESPRIT)-based method is proposed to estimate the gain–phase errors of both transmission and reception arrays and signal angles in bistatic MIMO radars, in which both transmitter and receiver are equipped with uniform linear array, and the first two sensors of transmit array and receive array are well calibrated to obtain a reference channel. In the field of angle estimation of array signal, the method of gain–phase error calibration is generally to ensure the consistency of the gain–phase characteristics of each channel; in contrast, there is no requirement of uniform gain–phase characteristics between the multi-transmitter array channels in an MSCI system.

In the SAR imaging field, a subspace algorithm of calibrating channel gain–phase errors for high-resolution and wide-swath (HRWS) SAR imaging is presented [12]. The proposed method is based on the fact that the signal subspace obtained from the eigendecomposition of covariance matrix equals the space spanned by the practical steering vectors. Channel gain–phase errors can be obtained through eigendecomposition of a special matrix which is the calculation result of the nominal steering vectors and the signal eigenvectors of the covariance matrix.

All the above methods on gain–phase errors calibration make use of the characteristics of eigen-subspace and estimate the gain and phase errors by matrix eigendecomposition. The basic feature of these methods is that the signals of multiple transmitting–receiving channels are separated during processing, but the received echoes are not separated by multiple channels in MSCI, so the channel gain–phase error calibration method based on subspace decomposition cannot be directly adopted in MSCI.

Without subspace decomposition, a method is proposed for joint SAR imaging and phase error correction in [13]. The problem is set up as an optimization problem in a non-quadratic regularization-based framework, and phase error correction is performed during the image formation process. The method involves an iterative algorithm, where each iteration includes consecutive steps of image formation and model error correction. A method for RCI with phase errors is proposed in [14], which adopts the sparse Bayes learning (SBL) framework and jointly estimates target scattering coefficients and phase error during the iterative steps. Soon after, in [15], a method is proposed for sparse auto-calibration for RCI with gain–phase errors(SACRCI), which transforms the imaging into the parameter estimation problem, and then estimates target scattering coefficient and gain–phase errors jointly. In [16], an auto-calibration expansion–compression variance-component (AC-ExCoV)-based auto-focusing method in a sparse Bayesian learning framework is proposed. These methods all take the gain–phase errors as unknown parameters and adopt an iterative procedure to jointly estimates target scattering coefficients and gain–phase errors. The targets in [15,16] are all sparse in small scenes. In other respects, the calibration of the gain–phase and synchronization errors is focused on for MSCI in [17], but a reference receiver is required to receive the direct signals of the transmitters to estimate the errors.

Considering large imaging scenes in MSCI, which means a large number of grid cells, results in very large computational complexity, limit the application for the above methods in large scenes. In [18,19], the problem of MSCI in a large scene is solved by dividing the large scene into strips. In [18], the echoes of the discrete clustered targets are detected to locate the strips with targets and only the regions of interest are discretized to a fine grid.

In order to solve the problem of MSCI with gain–phase error in a large scene, a method of MSCI based on strip-mode self-calibration of gain–phase errors is proposed in this paper. By dividing the target scene into strips, for each strip, the scattering coefficient and the gain–phase errors are combined into a multi-parameter optimization problem, which can be estimated by alternate iteration. Simultaneously, the gain–phase error estimation results of the previous strip can be carried into the next strip as the initial value. All strip imaging results, which can be obtained by correlated processing in turn, are spliced to obtain the image inversion results of the whole scene. To further improve gain–phase error estimation and imaging performance, after all the strips are processed in one round, the gain–phase error estimation results of the last strip can be taken as the initial value and substituted into the first strip for the correlated processing of the next round. In this way, all strips are processed in multiple rounds to obtain the final results.

The rest of the report is organized as follows. In Section 2, the strip-mode MSCI model with gain–phase errors is presented. Section 3 presents strip-mode MSCI algorithm with self-calibration of gain–phase errors. The analysis of the computation of the algorithm is discussed in Section 4. In Section 5, the performance of the proposed method is verified by numerical examples. Finally, Section 6 concludes this paper.

## 2. Strip-Mode MSCI with Gain–Phase Errors

**Figure 1:**
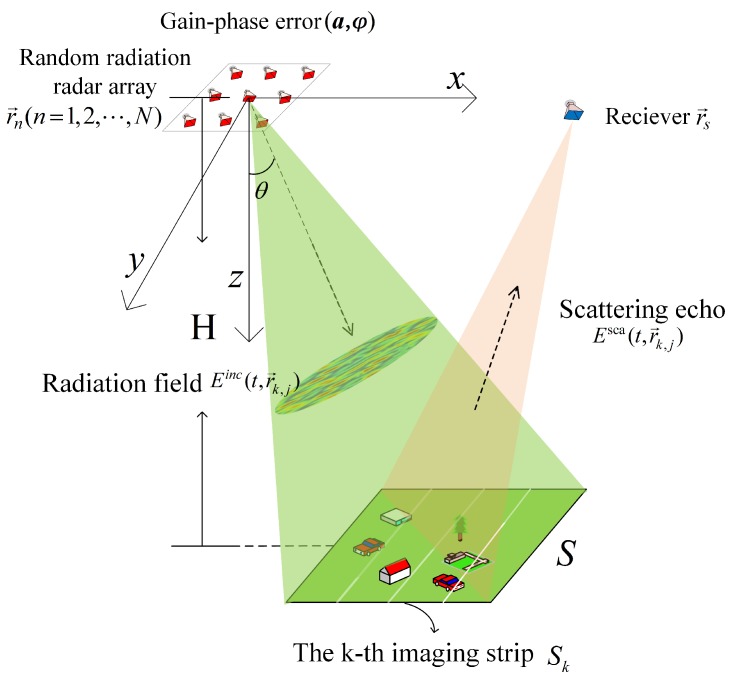
Geometry of MSCI.

As shown in Figure 1, a rectangular coordinate system is established with the center of the transmitting array as the origin; the MSCI system located in stationary platforms is composed of N transmitters and one receiver, whose position vectors are denoted as ${\vec{r}}_{n}$ and ${\vec{r}}_{s}$. The height of the transmitting array is *H* and θ is the squint angle. The independent narrow-pulse signals of random frequency hopping (RHF) which are transmitted synchronously by each antenna in multi-transmitter array can be expressed as:

(1) $$f_{n}\left(t\right)=\sum_{l=1}^{L}rect\left[\frac{t-\left(l-1\right)T_{p}}{\tau }\right]a_{n}A_{n}e^{j\varphi_{n}}\exp\left\{j2\pi f_{nl}\left[t-\left(l-1\right)T_{p}\right]\right\}$$

where $f_{nl}$ is its frequency of the *l*-th, l=1,2,⋯,L pulse emitted by the *n*-th, n=1,2,⋯,N transmitter, and randomly selected within the bandwidth *B*, and τ and $T_{p}$ is its narrow pulse width and period. $a_{n}A_{n}$ is the gain of the *n*-th transmitter, and $a_{n}$ denotes the gain error coefficient of the *n*-th transmitter, which equals 1 when there is no gain error, $\varphi_{n}$ denotes the phase error of the *n*-th transmitter, which equals 0 when there is no phase error. For simplicity, in the case that the bandwidth is narrow compared with the central frequency, we consider that the gain–phase errors are fixed in the imaging process.

According to the feature of radar range-gate, the random narrow pulse signals transmitted simultaneously by the multi-transmitter array can divide two-dimensional imaging area *S* into multiple different strips $S_{k},k=1\cdots K$ in the range direction [19]. The imaging strip $S_{k}$ has been divided into discrete J=P×Q grids, where *P* is the row number of azimuth resolution cells, and *Q* is the column number of range resolution cells, and position vectors of the center of *j*-th grid is denoted as ${\vec{r}}_{k,j}$, whose scattering coefficient is $\sigma \left(\vec{r_{k,j}}\right),j=1,2,\cdots ,J$. According to electromagnetic field propagation in free space, stochastic radiated fields at ${\vec{r}}_{k,j}$ in the *k*-th strip can be expressed as:

(2) Ekin(t,r→k,j)=∑n=1Nfnt−|r→k,j−r→n||r→k,j−r→n|cc4π|r→k,j−r→n|

The radiation field interacts with the *k*-th strip targets and the received signal of the *k*-th strip is:

(3) Eksca(t,r→k,j)=∑j=1J∑n=1Nfnt−|r→k,j−r→n|+|r→s−r→n||r→k,j−r→n|+|r→s−r→n|cc(4π)2|r→k,j−r→n||r→s−r→n|σ(r→k,j)=∑j=1JEkrad(t,r→k,j)σ(r→k,j)

Define the modified radiation filed of $S_{k}$ by considering the round-trip time of transmission after target reflection, which can be denoted as:

(4) $$E_{k}^{rad}\left(t,{\vec{r}}_{k,j}\right)=\sum_{i=1}^{N}\frac{f_{n}\left(t-\left(\left|{\vec{r}}_{k,j}-{\vec{r}}_{n}\right|+\left|{\vec{r}}_{s}-{\vec{r}}_{k,j}\right|\right)/c\right)}{{\left(4\pi \right)}^{2}\left|{\vec{r}}_{k,j}-{\vec{r}}_{n}\right|\left|{\vec{r}}_{s}-{\vec{r}}_{k,j}\right|}$$

The scattered echoes in strip-mode can be written as matrix vector:

(5) $$E_{k}^{sca}=E_{k}^{rad}\cdot \sigma_{k}$$

Because of the unknown gain–phase errors, the equation can be rewritten as:

(6) $$E_{k}^{sca}=E_{k}^{rad}\left(a,\varphi \right)\cdot \sigma_{k}$$

where $a={\left[a_{1},a_{2},\cdots ,a_{N}\right]}^{T}$ is the vector of the gain errors coefficient of the multi-transmitter array, and $\varphi ={\left[\varphi_{1},\varphi_{2},\cdots ,\varphi_{N}\right]}^{T}$ is the vector of the phase errors.

Strip-mode MSCI can obtain the target information ${\hat{\sigma }}_{k}$ in $S_{k}$ by the correlated processing between $E_{k}^{sca}$ and $E_{k}^{rad}$, which can be described as:

(7) $${\hat{\sigma }}_{k}=℘\left[E_{k}^{rad},E_{k}^{sca}\right]$$

where *℘* indicates the first-order correlated operator. Common correlated imaging algorithms include LS algorithm, TSVD regularization, Tikhonov regularization, TV regularization, sparse Bayesian learning, and etc.

Each strip is processed in turn to obtain all the MSCI results, and then all the strip images are spliced to obtain the whole scene imaging results. Since the reconstruction result is a one-dimensional vector deformed by the two-dimensional mesh of the strip, all the reconstruction results ${\hat{\sigma }}_{k}$ need to be converted into the corresponding two-dimensional form ${\hat{\sigma }}_{k}^{\prime }$. The imaging result of the whole scene can be expressed as:

(8) $${\hat{\sigma }}^{\prime }=\left[{\hat{\sigma }}_{1}^{\prime },{\hat{\sigma }}_{2}^{\prime },\cdots ,{\hat{\sigma }}_{K}^{\prime }\right]$$

**Figure 2:**
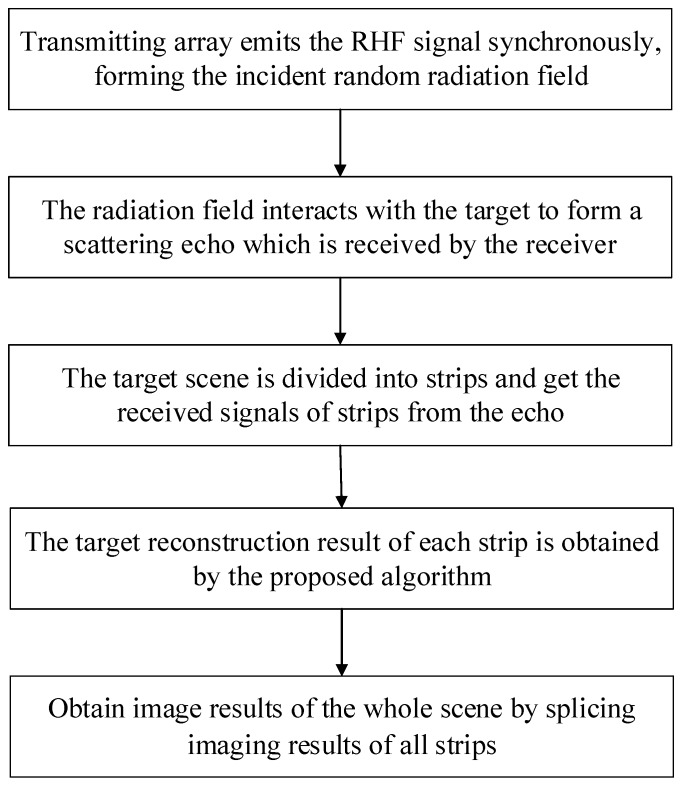
Imaging process flow chart.

The whole imaging process mainly includes: transmitting signal, interaction between radiation field and target to form scattering echo, receiving echo, dividing strip, MSCI with self-calibration of gain–phase errors of each strip, and obtaining image results of whole scene by splicing all strips’ imaging results. The flow chart of the whole imaging process is as Figure 2.

## 3. Strip-Mode MSCI Algorithm with Self-Calibration of Gain–Phase Errors

According to strip-mode MSCI method, the echo corresponding to each strip can be obtained from the received echo according to the distance gate. Therefore, the correlated imaging with gain–phase errors can be carried out separately for each strip. The modified radiation filed is unknown due to the gain–phase errors. The gain–phase error estimation and target reconstruction can be combined as an optimization problem, the cost function can be expressed as:

(9) $$F\left(\sigma_{k},a,\varphi \right)=\left|\right|E_{k}^{sca}-E_{k}^{rad}\left(a,\varphi \right){\left|\right|}_{2}^{2}+\lambda ||\sigma_{k}|{|}_{1}$$

where λ is the regularization parameter.

Then the k−th strip gain–phase errors calibration and target reconstruction can be converted into the following optimization problem:

(10) $$\left[\sigma_{k},a,\varphi \right]=\underset{\sigma_{k},a,\varphi }{\arg\min}F\left(\sigma_{k},a,\varphi \right)$$

In order to solve the above problems, a strip-mode MSCI algorithm based on self-calibration of gain–phase errors is proposed for the whole target scene. The algorithm is used to divide the whole scene into strips, and then the joint iterative solution of target reconstruction and gain–phase error estimation is carried out for each strip. In the process of one iteration, the target reconstruction results are obtained by minimizing of cost function through the given gain–phase errors. Then the gain–phase errors are estimated according to the target reconstruction results, and the modified radiation filed matrix is updated with the gain–phase error estimation for the next iteration. We terminate the iteration if ${∥\sigma_{k}^{i+1}-\sigma_{k}^{i}∥}_{2}^{2}/∥\sigma_{k}^{i}∥<\eta$ or the maximum number of iterations $I_{\max}$ is reached, where η is a predetermined threshold and the superscript *i* refers to the iteration. Key steps of the algorithm include target reconstruction and gain–phase error estimation.The concrete realization course of key steps is as follows.

### 3.1. Target Reconstruction

For a single strip, the target is reconstructed when the gain–phase errors is given. The initial gain–phase errors of the first strip a=1,φ=0. Target reconstruction can be expressed as:

(11) $$\sigma_{k}^{i+1}=\underset{\sigma_{k}}{\arg\min}\left|\right|E_{k}^{sac}-E_{k}^{rad}\left(a^{i},\varphi^{i}\right)\cdot \sigma_{k}^{}{\left|\right|}_{2}^{2}+\lambda ||\sigma_{k}^{}|{|}_{1}$$

The above formula is a standard compressed sensing reconstruction model. There are many existing methods for this problem, such as Basis pursuit (BP) algorithm [20], orthogonal matching pursuit (OMP) algorithm [21], Sparse Bayesian Learning (SBL) [22,23], etc. In this paper, OMP algorithm is adopted because it is simple in structure and easy to implement and analyze.

### 3.2. Gain–Phase Error Estimation

The gain and phase errors are estimated in an alternate iteration manner. The gain error is estimated as:

(12) $$a_{}^{i+1}=\underset{a}{\arg\min}\left|\right|E_{k}^{sca}-E_{k}^{rad}\left(a,\varphi^{i}\right)\cdot \sigma_{k}^{i+1}{\left|\right|}_{2}^{2}+\lambda ||\sigma_{k}^{i+1}|{|}_{1}$$

Since is ${∥\sigma_{k}^{i+1}∥}_{1}$ a constant in the iteration, Equation can be rewritten as:

(13) $$a_{}^{i+1}=\underset{a}{\arg\min}\left|\right|E_{k}^{sca}-E_{k}^{rad}\left(a,\varphi^{i}\right)\cdot \sigma_{k}^{i+1}{\left|\right|}_{2}^{2}$$

The above formula is a nonlinear least-squares problem, thus we use Newton’s method [24] to solve the problem.

Define $g_{k}\left(a,\varphi \right)={∥E_{k}^{sca}-E_{k}^{rad}\left(a,\varphi^{i}\right)\cdot \sigma_{k}^{i+1}∥}_{2}^{2}$, the updated $a^{i+1}$ estimation denoting by $a^{i}$ is computed as:

(14) $$a^{i+1}=a^{i}-{\left[\nabla_{a}^{2}g_{k}\left(a^{i},\varphi^{i}\right)\right]}^{-1}/\left[\nabla_{a}g_{k}\left(a^{i},\varphi^{i}\right)\right]$$

where $\nabla_{a}g_{k}\left(a^{i},\varphi^{i}\right)$ and $\nabla_{a}^{2}g_{k}\left(a^{i},\varphi^{i}\right)$ represent the gradient and Hessian with respect to the gain error respectively. After derivation and simplification, we have:

(15) ∇agk(ai,φi)=−2Re((Bk(ai,φi))Hw⌢)

(16) ∇a2gk(ai,φi)=2Re((Bk(ai,φi))HBk(ai,φi))

(17) $$\overset{⌢}{\;w}=E_{k}^{\mathrm{sca}}-E_{k}^{\mathrm{rad}}\left(a^{i},\varphi^{i}\right)\cdot \sigma_{k}^{i+1}$$

(18) $$B^{k}\left(a^{i},\varphi^{i}\right)=\left[b_{1}^{k}\left(a^{i},\varphi^{i}\right),\cdots b_{N}^{k}\left(a^{i},\varphi^{i}\right)\right]$$

where Re() denotes the real part,

(19) $$b_{n}^{k}\left(a^{i},\varphi^{i}\right)=e^{j\varphi_{n}^{i}}\left[\begin{array}{ccc} S_{n}\left(t_{1},{\vec{r}}_{k,1}\right) & \cdots & S_{n}\left(t_{1},{\vec{r}}_{k,J}\right)\\ \vdots & \ddots & \vdots \\ S_{n}\left(t_{L},{\vec{r}}_{k,1}\right) & \cdots & S_{n}\left(t_{L},{\vec{r}}_{k,J}\right) \end{array}\right]\cdot \sigma_{k}^{i+1}$$

(20) Sn(t,r→k,j)=f^nt−|r→k,j−r→n|+|r→s−r→k,j||r→k,j−r→n|+|r→s−r→k,j|cc(4π)2|r→k,j−r→n||r→s−r→k,j|

(21) $${\hat{f}}_{n}\left(t\right)=\sum_{l=1}^{L}rect\left[\frac{t-\left(l-1\right)T_{p}}{\tau }\right]A_{n}\exp\left\{j2\pi f_{nl}\left[t-\left(l-1\right)T_{p}\right]\right\}$$

In the same way, the phase error is estimated as:

(22) $$\begin{array}{c} \varphi^{i+1}=\underset{\varphi }{\arg\min}\left|\right|E_{k}^{sca}-E_{k}^{rad}\left(a^{i+1},\varphi \right)\cdot \sigma_{k}^{i+1}{\left|\right|}_{2}^{2}+\lambda ||\sigma_{k}^{i+1}|{|}_{1} \end{array}$$

The updated $\varphi^{i+1}$ estimation denoting by $\varphi^{i}$ is computed as:

(23) $$\varphi^{i+1}=\varphi^{i}-{\left[\nabla_{\varphi }^{2}g_{k}\left(a^{i+1},\varphi^{i}\right)\right]}^{-1}/\left[\nabla_{\varphi }g_{k}\left(a^{i+1},\varphi^{i}\right)\right]$$

The gradient and Hessian with respect to the phase error can be computed as:

(24) ∇φgk(ai+1,φi)=−2Im((Dk(ai+1,φi))Hw⌢)

(25) ∇φ2gk(ai+1,φi)=2diag(Re((Dk(ai+1,φi))Hw⌢))+2Re((Dk(ai+1,φi))HDk(ai+1,φi))

(26) $$D^{k}\left(a^{i},\varphi^{i}\right)=\left[d_{1}^{k}\left(a^{i},\varphi^{i}\right),\cdots d_{N}^{k}\left(a^{i},\varphi^{i}\right)\right]$$

where Im() denotes the imaginary part, diag() is the diagonalization operation.

(27) $$\begin{array}{c} d_{n}^{k}\left(a^{i},\varphi^{i}\right)=e^{j\varphi_{n}^{i}}a_{n}^{i+1}\left[\begin{array}{ccc} S_{n}\left(t_{1},{\vec{r}}_{k,1}\right) & \cdots & S_{n}\left(t_{1},{\vec{r}}_{k,J}\right)\\ \vdots & \ddots & \vdots \\ S_{n}\left(t_{L},{\vec{r}}_{k,1}\right) & \cdots & S_{n}\left(t_{L},{\vec{r}}_{k,J}\right) \end{array}\right]\cdot \sigma_{k}^{i+1} \end{array}$$

The above is about the single iteration process of gain–phase error estimation by Newton’s method. In the i−th iteration, $\left(a^{i},\varphi^{i}\right)$ will be updated to $\left(a^{i+1},\varphi^{i+1}\right)$. The initial gain–phase errors of the first strip a=1,φ=0. The gain–phase error estimation results of the former strip are taken as the initial value and substituted into the latter strip, which makes the estimation results of the latter strip more accurate. After the first round of correlated processing with calibration of gain–phase error of all strips is completed, the gain–phase error estimation results of the last strip are brought into the first strip for the next round, and the whole imaging area divided into strips is processed in multiple rounds to obtain the final results.

The whole process of the algorithm is as follows:
**Algorithm 1:** Strip-Mode MSCI Algorithm with Self-Calibration of Gain–phase Errors
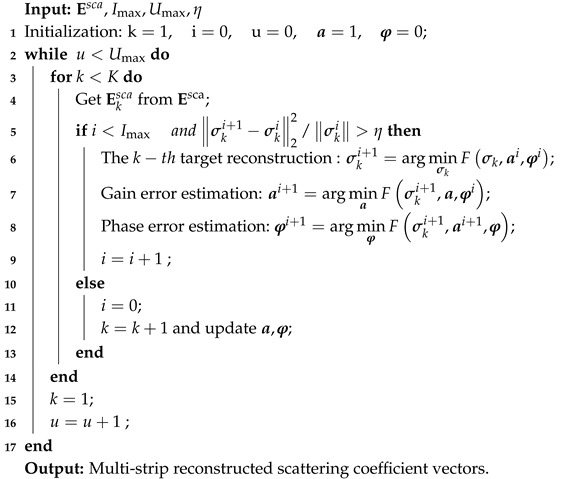


## 4. Analysis

The proposed strip-mode MSCI method based on self-calibration of gain–phase errors can greatly reduce the computational cost of the imaging process. The total grid number of the target scene is *M*, divided into *K* strips, and the number of grid in each strip is *J*. The main operations of an iteration during imaging process include updating the modified radiation filed matrix, target reconstruction, gain–phase error estimation by Newton’s method. According to the characteristics of MSCI, generally the narrow pulse number *L* should satisfy L>M. Compared to no strip division, after the target scene is divided into *K* strips, the number of grids with in each strip is decreased to M/K, the number of narrow pulse is decreased to L/K, so the scale of the modified radiation filed matrix is reduced to $ML/K^{2}$. Therefore, the computation required for updating the modified radiation filed matrix and Newton’s method is reduced significantly. For the OMP algorithm in the target reconstruction process, in the case that the sparsity is *d*, the computation is O(d·L·M) [21] when there are no strips, in contrast, when dividing into *K* bands, the computation is K·O(d/K·L/K·M/K). The above discussion is about the change of the computation in an iteration. In the actual process, due to the strip division, the target scene and the operation process are simplified, the average number of iterations required in the imaging process is also decreased, and the operation time is further reduced.

## 5. Simulations

The effectiveness of proposed method is verified by several simulations in this section. An X-band MSCI radar system with center frequency 10 GHz is considered. The scenario for simulation is shown in Figure 1. The height of the transmitter array is 300 m, which consists of 25 elements to form a uniform array of 3 × 3 m in size. The distance of target scene is 450 m, and the size of target scene is discretized into 40 × 40 grids with grid size of 2 × 2 m. We initialize $a=1,\varphi =0,I_{\max}=100,\eta =10^{-4}$. Some system parameters are given in Table 1, and the parameters of gain–phase errors are given in Table 2.

### 5.1. Performances Under Different Number of Strips

In this subsection, simulations are taken to compare the performances with different strips. The normalized mean square error (NMSE) is used to quantify the reconstruction effect and gain–phase error estimation, with the definition as: NMSEdB=20lg(x^−x2x^−x2xx2), where x denotes the target imaging or gain–phase errors, accordingly, $\hat{x}$ denotes the target reconstruction or gain–phase error estimation results.

**Figure 3:**
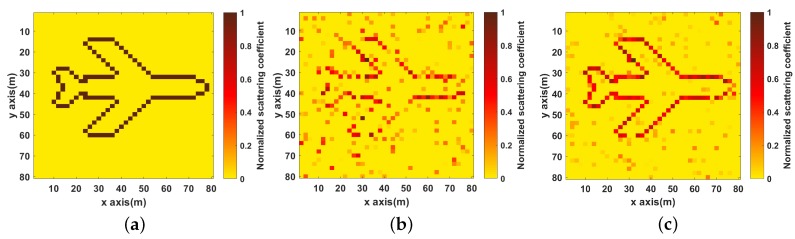

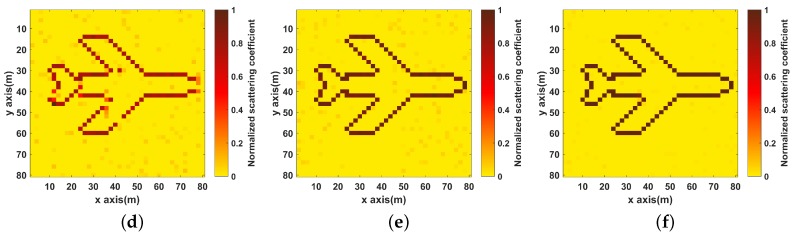
Imaging results (**a**) objective model; (**b**) Imaging results of OMP; (**c**–**f**) Imaging results under different number of strips (**c**) no strip; (**d**) two strips; (**e**) four strips; (**f**) eight strips.

**Figure 4:**
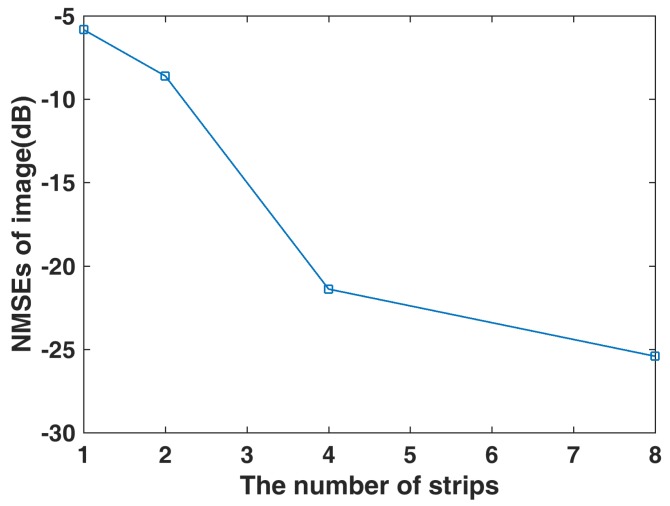
NMSE of target reconstructions under different number of strips.

It can be seen in Figure 3b that the image is defocused and many spurious scatterers exist with for the OMP algorithm. In Figure 3c–f, it can be seen that the image become clearer and clearer with increase in the number of strips. The NMSEs of the reconstruction images under different strips are given in Figure 4, and it shows that NMSEs are decreased as the number of strips increases, which means the quality of imaging is getting better. Compared to no strip, proposed method with eight strips improves the imaging performance by about 20 dB from the NMSE perspective.

**Figure 5:**
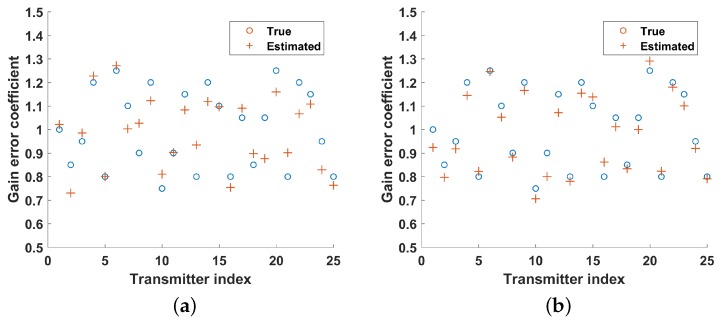

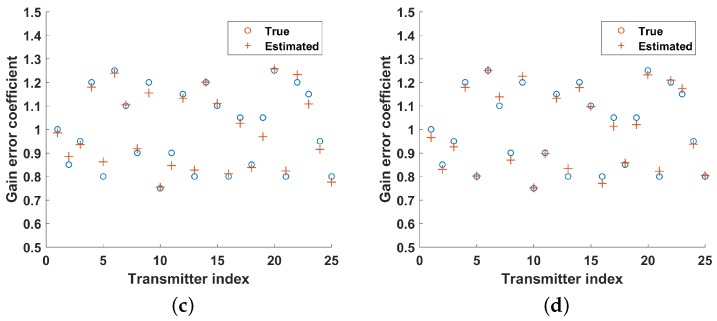
Gain error estimation under different number of strips (**a**) no strip; (**b**) two strips; (**c**) four strips; (**d**) eight strips.

**Figure 6:**
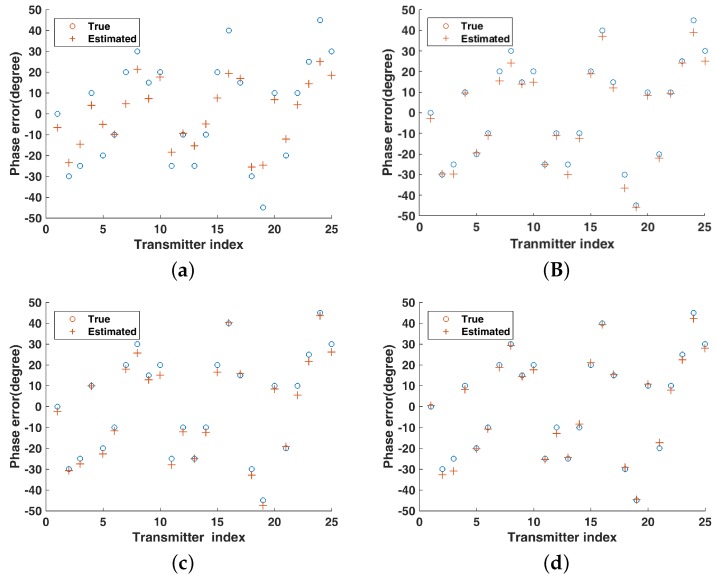
Phase error estimation under different number of strips (**a**) no strip; (**b**) two strips; (**c**) four strips; (**d**) eight strips.

**Figure 7:**
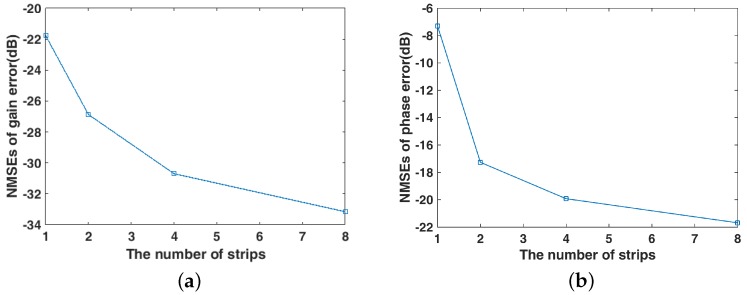
Gain–phase error estimation performance under different strips (**a**) NMSE of gain error estimation; (**b**) NMSE of phase error estimation.

In Figure 5 and Figure 6, it can be seen that the estimates of gain and phase error are closer to the actual value as the number of strips increases. As shown in Figure 7, the NMSEs of gain–phase error estimation are getting lower as the number of strips increases, which means estimation errors are getting lower, and it is proved that the proposed method in this paper can improve the accuracy of gain–phase error estimation effectively.

**Figure 8:**
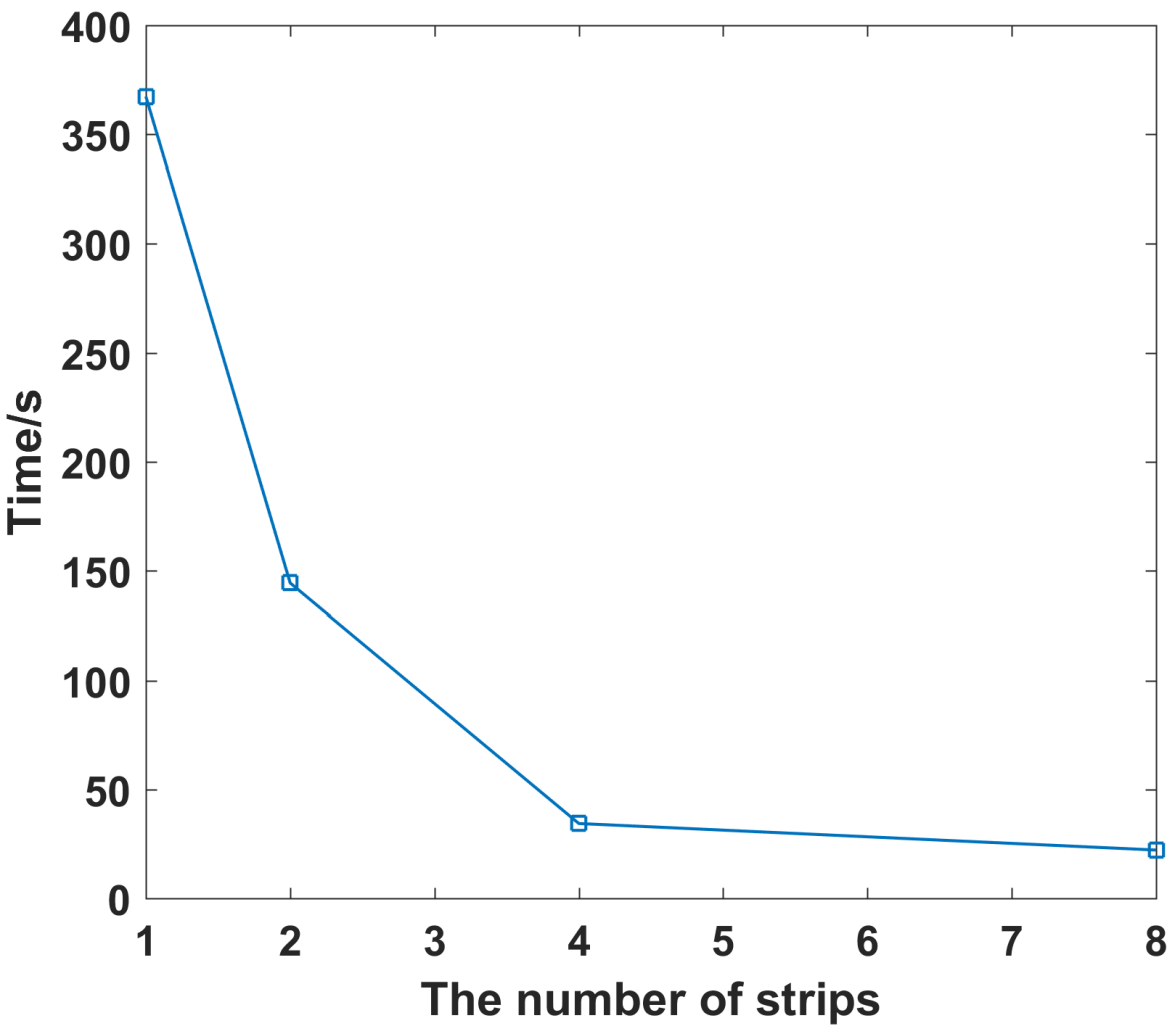
Imaging time under different number of strips.

In Figure 8, as the strip increases, the imaging time decreases significantly, which is consistent with the analysis in this paper. It takes less than 1/15 of time by divided into eight strips compared with no strip. It is proved that the strip division can greatly reduce the time required for the correlated imaging process.

### 5.2. Performance under Different SNRs

**Figure 9:**
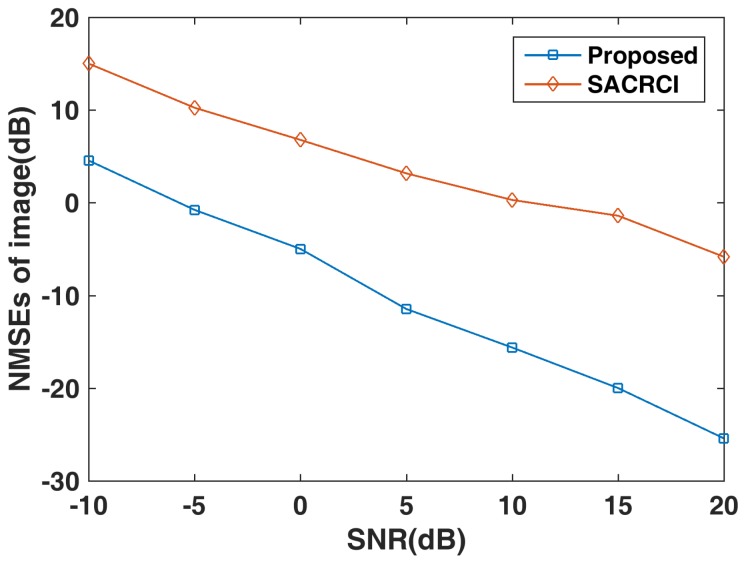
NMSE of target reconstructions under different SNRs.

**Figure 10:**
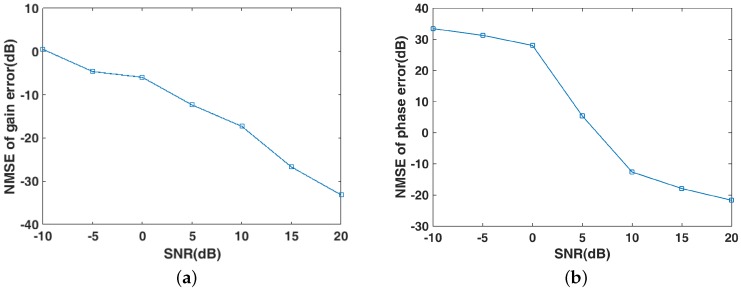
Gain–phase error estimation performance under different SNRs (**a**) NMSE of gain error estimation; (**b**) NMSE of phase error estimation.

In this subsection, we compare the performance of algorithms under different SNRs, for the proposed method and SACRCI [15]. As shown in Figure 9, the imaging quality is improved significantly as the SNR increases, which means the two method are sensitive to noise. The proposed method improves the imaging performance by more than 10 dB compared with SACRCI from the NMSE perspective. In Figure 10, it can be seen that the gain–phase error estimation is also sensitive to noise.

### 5.3. Performance under Different Transmitting Array Configurations

**Figure 11:**
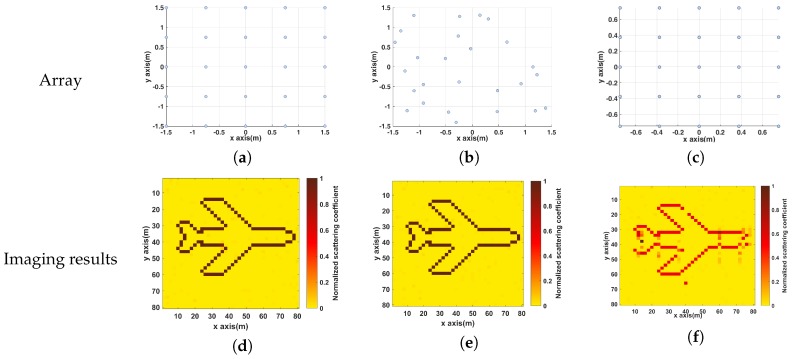
Imaging results under different transmitting array configurations (**a**–**c**) Different transmitting array configurations; (**d**–**f**) Imaging results.

In MSCI, transmitting array configurations can influence imaging effect, and considering this, we perform simulations in this subsection to compare the performance under different transmitting array configurations. In Figure 11a, the transmitting array is a array with its aperture of 3 m. In Figure 11b, the array elements are randomly distributed on the plane. In Figure 11c, the aperture of the uniform planar array is reduced to 1.5 m. From the imaging results, it can be seen that the size of the array aperture influences the target reconstruction significantly, which is consistent with the relationship between the array aperture size and the imaging resolution.

### 5.4. Performance under Different Center Frequencies

**Figure 12:**
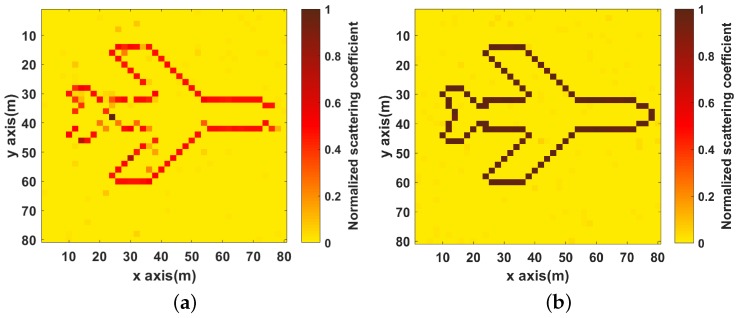
Imaging results under different center frequencies (**a**) 1 GHz; (**b**) 40 GHz.

In this subsection, performance under different center frequencies is compared by simulations. I can be seen in Figure 12 that target reconstruction result is not clear when center frequency is 1 GHz, in contrast, when center frequency is 40 GHz, the target reconstruction effect is much better. This is because the resolution of MSCI is related to the center frequency, the higher the center frequency, the better the resolution, and the better the imaging effect under the same grid division.

It can be seen in Figure 12 that the target reconstruction result is not clear when center frequency is 1 GHz; in contrast, when center frequency is 40 GHz, the target reconstruction effect is much better. This is because the resolution of MSCI is related to the center frequency, the higher the center frequency, the better the resolution, and the better the imaging effect under the same grid division.

### 5.5. Performance under Different Target Scenes

Since Target reconstruction results is obtained by OMP, the reconstruction performance may be affected by the target, more precisely, the sparsity of target. In this subsection, we design simulations to compare the performance under different target scenes.

**Figure 13:**
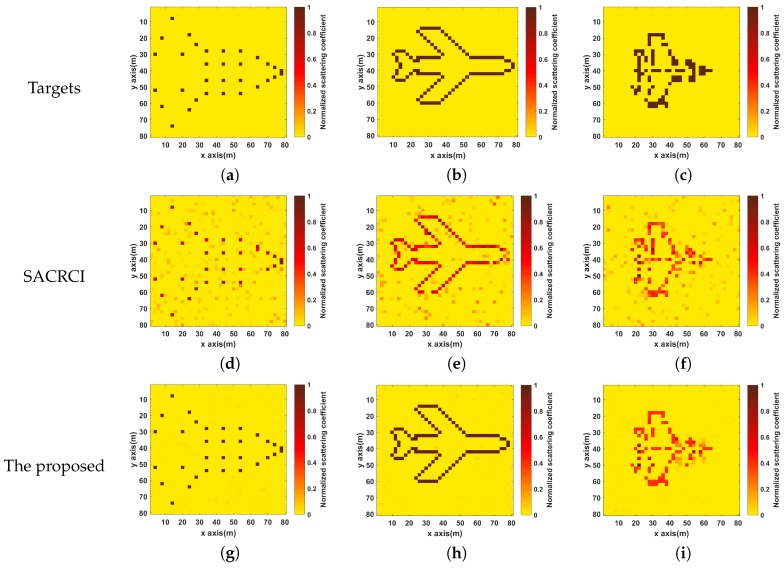
Imaging results for different target scenes (**a**–**c**) Three different target scenes; (**d**–**f**) Imaging results of SACRCI; (**g**–**i**) Imaging results of the proposed method.

As shown in Figure 13a–c are three different target scenes. It can be seen that the images become blurred as the complexity of targets increases, which means the less sparse target would make the target reconstruction more difficult and the gain–phase error estimation performance is also affected. Comparing with the results obtained by SACRCI, the spurious scatterers in the bottom three images which are obtained by the proposed method, are much less, and the three targets are identified clearly. It proves that the proposed method can improve the imaging performance by reducing the complexity of correlated imaging processing.

### 5.6. Discussion

Lots of numerical simulations validate potential advantages of the proposed method to shorten the imaging time dramatically and improve the imaging and gain–phase error estimation performance, and show the performance under different SNRs, different targets, different array configurations and different center frequencies. In the actual system, since the proposed method uses the range-gate characteristic of the narrow pulse to divide the imaging area into strips, the transmitting system must have a high rectangular coefficient, and each transmitting element needs a high-precision time-frequency reference. The system must have a high-precision time-frequency synchronization to ensure the accurate separation of the corresponding parts of each strip from the echo. These are great challenges in actual MSCI system.

## 6. Conclusions

This paper proposes a method of MSCI based on strip-mode self-calibration of gain–phase errors. By dividing the target scene into strips, the target reconstruction and the gain–phase error estimation are solved simultaneously by alternate iteration. By simulations it can be seen that the gain–phase errors calibration and imaging effect have been greatly improved and the time required for the entire imaging process has been greatly shortened. Moreover, to improve imaging and gain–phase error estimation performance furtherly, not only are the gain–phase error estimation results of the previous strip carried into the next strip as the initial value, but also the gain–phase error estimation results of the last strip are the initial value in next round. In conclusion, the proposed method can greatly reduce the time required by the imaging process and improve the imaging quality, so it can rapidly achieve gain–phase errors calibration and target imaging in a large scene. 

References

## Figures and Tables

**Table 1:** System parameters.

Parameter	Value
Center Frequency	10 GHz
Bandwidth	500 MHz
Transmitting signal mode	Frequency hopping
Number of transmitters	25
Aperture of transmitter array	3 × 3 m
Imaging grids	40 × 40
Size of imaging grids	2 × 2 m
Height of transmitter array	300 m
The squint angle	$45^{\circ }$

**Table 2:** Gain–phase error parameters.

Index	1	2	3	4	5	6	7	8	9	10	11	12
a	1	0.85	0.95	1.20	0.80	1.25	1.10	0.90	1.20	0.75	0.90	1.15
φ/∘	0	−30	−25	10	−20	−10	20	30	15	20	−25	−10
**13**	**14**	**15**	**16**	**17**	**18**	**19**	**20**	**21**	**22**	**23**	**24**	**25**
0.80	1.20	1.10	0.80	1.05	0.85	1.05	1.25	0.80	1.20	1.15	0.95	0.80
−25	−10	20	40	15	−30	−45	10	−20	10	25	45	30
